# Anxiety and Psycho-Physiological Stress Response to Competitive Sport Exercise

**DOI:** 10.3389/fpsyg.2018.01469

**Published:** 2018-08-27

**Authors:** Gaelle Tanguy, Emmanuel Sagui, Zagnoli Fabien, Charles Martin-Krumm, Frédéric Canini, Marion Trousselard

**Affiliations:** ^1^Service de Neurologie, Hôpital d’Instruction des Armées Laveran, Marseille, France; ^2^Ecole du Val de Grâce, Paris, France; ^3^Fed 3C, LNC, UMR 7291, Aix Marseille Université, Marseille, France; ^4^Service de Neurologie, Hôpital d’Instruction des Armées Clermont-Tonnerre, Brest, France; ^5^Unité de Neurophysiologie du Stress, Département de Neurosciences et Contraintes Opérationnelles, Institut de Recherche Biomédicale des Armées, Brétigny-sur-Orge, France; ^6^Laboratoire de Psychologie de l’Ecole de Psychologues Praticiens de Paris, Paris, France; ^7^APEMAC, EA 4360, EPSaM, Université de Lorraine, Nancy, France; ^8^Chaire Mindfulness, Bien-être au Travail et Paix Économique, Grenoble Ecole de Management, Grenoble, France

**Keywords:** anxiety management, stress, selective physical exercise, special forces, overtraining

## Abstract

**Introduction:** Sport is recognized as beneficial for health. In certain situation of practice, it nevertheless appears likely to induce a stress response. Anxiety is a stress response-modulating factor. Our objective is to characterize the role of anxiety in the stress response induced by a selective physical exercise.

**Method:** Sixty-three young male military conducted a selective sporting running event (a 8-km commando-walk) and were recorded the day before, the day of the race, and the day after. The variables were psychometric [personality questionnaires, coping and anxious/stress state, and physiological (nocturnal heart rate variability and actigraphy)]. The subjects were classified, using scores on anxiety questionnaires at baseline, into two groups according to their anxious (G ANX) or non-anxious (G N-ANX).

**Results:** Before the race, the G ANX was characterized by a lower level of self-esteem, higher scores in dysfunctional coping and a greater perceived stress compared to the G N-ANX. Compared to G N-ANX, the stress response to the exercise was higher in G ANX: G ANX exhibited (Selye, 1950) in immediate post-exercise, greater level in activation markers, and mental fatigue associated with a same level of physical fatigue and (Kim et al., 2018) in nocturnal post-exercise, an increase in sympathetic activation associated with a higher sleep fragmentation.

**Conclusion:** A competition selection sport exercise causes a stress response, particularly for anxious subjects. Anxious status could be involved in the risk of emergence of overtraining in sport practice. These results must be taken into account when sport practice is used for anxiety management.

## Introduction

Stress is a non-specific and complex response of a human body submitted to a stressor, which responds to an adaptive function. It is described as the general adaptation syndrome (GAS) (Selye, 1950) and is divided into three stages: an initial alarm stage, followed by a resistance stage to the stressor, which lasts and to which the human body has to adapt to, and lastly a recovery stage. The stressor characterizes any situation activating the stress pathway, regardless its nature, its depth and its duration (Selye, 1950). It can be external to the subject, imposed by an environmental change, or auto-generated by affects or negative-valence thoughts, especially anxious thoughts.

From a physiological point of view, if we can artificially consider all the stress players separately in regard to their very nature, it is important to consider them within their dynamics and their interactions. Strictly speaking, stress corresponds to the activation of the catabolic mechanisms: the activation of the corticotropic axis and of the sympathetic autonomic nervous system (ANS) and withdrawal of the parasympathetic ANS. The sympathetic ANS prepares the body to action when facing a stressor thanks to an increased mobilization of the energy resources of the body in order to support the alert reaction (flight or fight) and attention to the world. The corticotropic axis facilitates the availability of the body energy resources over time. Recovery is possible through anabolic pathways entailing in particular sleep and activation of the parasympathetic ANS. These pathways represent the link between the central nervous system and the periphery, allowing the body to act in a coordinated and adjusted manner. They allow the rating of the level of stress response of a body by peripheral physiological measurements.

The heart rate variability dynamically informs about the regulation of the balance between the sympathetic and the parasympathetic ANS (Task Force of the European Society of Cardiology and the North American Society of Pacing and Electrophysiology, 1996; Kim et al., 2018). When it is well-regulated (eustress), the stress expresses a physiological mechanism managing acute and chronic biological costs. A burnout situation will result if the stressor is too intensive and/or too long, or else if the stressed individual’s response capacities are not adapted (distress). There is a strong inter-individual variability in the psychobiological reaction to a stressor, and it is identical within the same individual for the physical or psychic stressors (McEwen, 2007). The stress response, whatever the stressor, forms part of an earthly body whose limits are influenced by the genome and the history of the subject. These factors inherent to each subject constitute an endogenous limitation, which expresses the more or less big efficiency of the biological systems to cope with the demands imposed by the stressors. This biological efficiency is modulated by the *psyche* that is going to deal with an event as a stressor as soon as it is perceived as new, unpredictable, or else uncontrollable. As soon as an individual assigns one or several of these characteristics to an event, he perceives his/her resources as inappropriate to the perception he has of the coercion (Lazarus, 1993). The psychological factors participating in this inter-individual variability belong to two main categories: the moderators, which determine whether or not the response to stress will emerge (the most studied are the personality traits) (Baron and Kenny, 1986), and the mediators, which are going to modulate this response once it is implemented (we also speak about adjusting factors). Among these psychological factors, anxiety plays a major part. As for any psychic dimension, the trait (personality, moderator factor) must be distinguished from the state and the mood (mediator factor; Bolmont and Abraini, 2001). The anxiety trait is considered as a relatively stable emotional disorder that characterizes a personality manifesting through a sense of insecurity (Spielberger and Smith, 1966). The anxiety-state is a fear characterized by behavioral, physiological, and cognitive responses, which encompass those of stress but do not stop at them. The individuals with an anxious personality are particularly sensitive to emotional stimuli with a negative valence, which contribute to the risk of developing anxious pathologies. This sensitivity contributes to reducing the adaptation of anxious individuals to stressors. The anxiety trait is therefore considered as a moderator fostering the emergence of important and repeated stress reactions. The anxiety-state reflects a time period focused to anxious feeling relatively to a present or future meaningful context (Hainaut and Bolmont, 2006). So, if the anxious trait empirically allows to prejudge an anxious state, the opposite is less systematic as a result of the anxious state depending on its context. The anxiety context would express a dynamic emotional process that could include an anxious emotion or an anxious mood depending on its intensity and duration. The anxious state is considered as a stress reaction mediator positioning the perception of context as a *per se* actor of the dynamic to stress response. Numerous managements of anxiety do exist in terms of prevention and/or treatment. Practicing a physical activity, aerobic or anaerobic, has been put forward for a long time as an ecological method to reduce the anxiety-state whatever the level of anxiety-trait (Petruzzello et al., 1991; Anderson and Shivakuvar, 2013). Furthermore, the physical activity is of undeniable interest for the prevention and treatment of mental diseases in relation to anxiety (Anderson and Shivakuvar, 2013; Kumar, 2017). Beyond this, somatic benefits do exist, especially cardiovascular effects are benefits (Paffenbarger et al., 1986). It also plays a beneficial role on the stress-related diseases. These benefits are all the more important when the practice is regular (Petruzzello et al., 1991). The WHO recommendations suggest to practice a moderate activity (at least 3 h a week) or an intense activity (at least 20 min three times a week). However, some sport activities generate deleterious stress responses. In the context of a compulsory practice, the physical activity turns up to act as a stressor for the individual. Preclinical studies on rats undergoing rehabilitation show a stress response, which delays recovery when the rehabilitation exercises are compulsorily done (Ke et al., 2011). As far as humans are concerned, a compulsory sport practice also triggers a stress reaction (Nicolas, 2009; Ke et al., 2011). In the context of a regular practice, 13% of the sportsmen develop a state ranging from exhaustion to overtraining, and 7.8% of the military are addicted to sport (Tello et al., 2011). A need for performing a more-and-more frequent and more-and-more intense physical activity is observed, particularly among sportsmen characterized by a high anxiety-trait level (Cook and Hausenblas, 2008). In the context of competition, an anxiety is often present following a sports contest (Shabnam Hamidia, 2010). It is fostered by the existence of an anxiety-trait, -state and/or -mood and of a strong social desirability (Craft et al., 2003; Shabnam Hamidia, 2010). Finally, after a competitive effort, compared to a similar effort in a training context, a longer physiological recovery is observed, resulting in the persistence of a high sympathetic tone (Foster, 1998). Thus, practicing a physical activity in the context of an external and/or internal compulsory practice induces a stress response likely to be potentiated among the anxious sportsmen. This response could have consequences in terms of recovery among these patients. These data suggest that certain sport practices conditions might not be beneficial to the health, especially among anxious patients.

The military environment provides a model to explore these issues. As part of the army forces retention, the military institution imposes a regular physical activity practice to its staff for a minimum of 8 h a week. In this sense, soldiers are professional sportsmen. The purpose of this training is twofold: toughening the soldiers for the future struggles, but also preparing them to sports events selection, among which the commando march is the most common selection procedure used. This paradigm characterizes a selective physical exercise, intense in duration and in “intensity,” which forms part of well-codified regular practice. It is legitimate under these elements to consider that the military sports selection is similar to competitions) and that it constitutes a practice setting likely to generate a stress response, especially among anxious staff members. We wish to study the psycho-physiological stress response in the military individual compelled to a selective commando march. The purpose of this study is to assess the impact of the anxious status on the dynamic of stress response following the commando march.

## Materials and Methods

### Participants

Sixty-three voluntary French male soldiers (age: 20.3 ± 2 years; BMI 23.4 ± 2.3) were included in a prospective study. All were in initial training for the fusilier commando specialty, a French navy special force unit. The data collection was carried out on four commando training-courses that took place between May 2012 and January 2013. Three individuals were excluded from the analysis due to missing data; the individuals had not completed the submitted questionnaires. Chart 1 sums up the characteristics of these training-courses. This study was agreed by the Ouest six persons protection comity under the reference 2011-A01660-41. All the prospective subjects were informed about the conduct of this study and gave their written consent prior to their participation.

### Physical Test

The experimental paradigm is an 8-km commando march in the minimum amount of time. This physical test specific to the military environment consists in a race dressed in combat gear with “rangers” type of shoes carrying an 11-kg backpack. As regards a qualifying event, the subjects were to perform the best possible time, the time for this event interfering in their ranking and in their final selection for the fusilier commando specialty. None of the subjects had already the experience of this event.

### Physiological Variables

#### Night Heart Rate Variability

The heart rate was measured, thanks to a heart rate monitor (ActiHeart^®^, CE). It is a small (35 mm × 35 mm × 15 mm), light (16 g) self-adhesive device placed on the chest and connected to two self-adhesive ECG electrodes causing no discomfort. To isolate specifically the responsiveness of the sympathovagal balance, the calculation of the LF/HF (Low Frequency/High Frequency) ratio, was chosen as variable of interest (Kubios^®^ software). The higher the value is, the more important the sympathetic activation is.

#### The Quality of Sleep

The quality of sleep is assessed thanks to a wrist actimeter (Wellness Wireless Watch VIVAGO EU644534000). The devices are light watches causing no inconvenience for the activities. The processed variables are the time asleep, the sleep efficiency, the sleep onset latency, and the sleep fragmentation (Wellness Wireless VIVAGO^®^ software).

### Psychological Variables

Three questionnaires have assessed the psychological dispositions (trait). The anxious personality was assessed by the anxiety-trait Spielberger questionnaire (State-Trait-Anxiety Inventory; STAI Y-B) (Spielberger, 1983). This self-assessment questionnaire consists of 20 items. For each item, the subject must indicate if he characterizes his usual anxious feeling from a Likert scale ranging from 1 (no) to 4 (yes). The average value is 41.9 ± 9.5 in a French population. A score greater than or equal to 47 is considered as being pathological (Bruchon-Schweitzer and Paulhan, 1993).

The self-esteem has been measured, thanks to the Rosenberg Self Esteem Scale (SES) (Rosenberg, 1979). This self-assessment scale measures, thanks to 10 items, the opinion one has about oneself, in a non-specific way. For each of the items, the subject must indicate if he characterizes his feeling from a Likert scale ranging from 1 (totally disagree) to 4 (totally agree). A score of less than 15 is considered as assessing a low self-esteem; a score between 15 and 25 is considered as assessing a normal self-esteem.

The stress adjustment, or “coping,” has been measured by the Coping Inventory of Stressful Situations (CISS) (Endler and Parker, 1999). This questionnaire assesses via 48 items the way the subject usually copes with stresses. It allows to assess the subject in the three types of coping: the task focused coping, the emotion focused coping, and the passive coping (avoidance, social distraction, and diversion). The average value in the population is of 58.6 ± 10 for the task focused coping, 39.2 ± 11.5 for the emotion focused coping, 38.1 ± 9 for the avoidance focused coping, 17.5 ± 5.5 for the distraction focused coping, and 13.3 ± 4.1 for the social diversion type coping (Trousselard et al., 2010).

#### Three Questionnaires Assess the Psychological State

The anxious state was assessed through the Spielberger’s state-anxiety questionnaire (STAI Y-A). This self-questionnaire includes 20 items. For each of these items, the subject must indicate if he characterizes his anxious feeling while filling in the questionnaire from a Likert’s scale ranging from 1 (no) to 4 (yes). The average value is of 35.7 ± 10.3 in a French population. An equal or higher score than 41 is considered as being high (Bruchon-Schweitzer and Paulhan, 1993).

The subject’s mood was measured by the *Profile of Mood State* (POMS) (Shacham, 1983). This self-questionnaire assesses, by the means of 37 adjectives, the different states of mood of a subject and their fluctuation (repetition of questionnaires). The subject must indicate how this adjective reflects his state from a Likert’s scale ranging from 1 (not at all) to 4 (extremely). The adjectives gather in six factors defining six states of mood, which are anxiety, depression, confusion, anger, fatigue, and vigor. This questionnaire, assessing the state of mood of a subject at the moment when he answers the different items, has a transitional value.

The perceived stress was assessed through Cohen’s perceived stress scale [*Perceived Stress Scale* (PSS)] (Cohen et al., 1983). This self-questionnaire includes 14 items. For each of the items, the subject must indicate his perception of stress while filling it in from a Likert’s scale ranging from 1 (never) to 4 (often).

### Experimental Procedure

**FIGURE 1:**
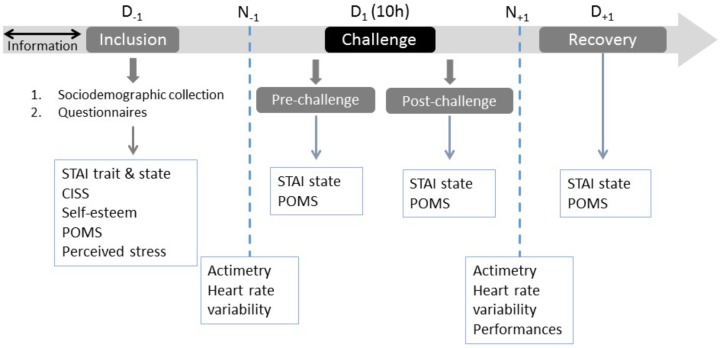
Experimental protocol with the distribution of the four sessions: day before the challenge (inclusion session; D_-1_), morning just before the challenge (pre-challenge session; pre-challenge D_1_), end of the challenge (post-challenge session; post-challenge D_1_), and day after the challenge (recovery session; D_+1_).

Each of the trainings followed an identical protocol including four sessions and was organized around the commando march challenge, which systematically started at 10 a.m. **Figure 1** sums up the experimental protocol with the distribution of the four sessions over time: at 5 p.m. on the day before the challenge (inclusion session; D-1), in the morning just before the challenge (pre-challenge session; prechallenge D1), at the end of the challenge (post-challenge session; post-challenge D1), and at 9 a.m. on the day after the challenge (recovery session; D+1).

All fusilier commando training, participants benefited from a medical examination during the week before the beginning of the training assessing their medical fitness to do it. On D-1, the subjects filled in the following questionnaires: socio-biographic and sports practice, self-esteem (EES), anxiety-trait and -state (STAI Y-A and-B), mood (POMS), coping (CISS), perceived stress (PSS). During the D1 pre-challenge and post-challenge sessions and D+1, the subjects filled in the anxiety-state and mood questionnaires.

The heart rate variability and actimetrics data were collected in *N* - 1 and *N* + 1 on a recording time of 6 h for all subjects. This period was established within an 11 p.m. to 5 a.m. timetable corresponding to the moments when the subjects were all in bed.

### Statistical Analysis

The data were registered under Excel 2010 (Microsoft^®^, Redmond, WA, United States) and analyzed with Statistica V7.1 (Statsoft^®^, France). The discrete variables were compared through the Chi^2^ test or through the Fisher’s exact test when the conditions for the Chi^2^ use were not completed. The continuous variables, presented by their average ± standard deviation, were compared by variance analyses (ANOVAs). A principal components analysis pre-treatment was conducted in order to assess the relevance of the anxiety psychometric data reduction collected in the inclusion (anxiety-trait, -state, -mood baselines) with less descriptors. The results of the analysis isolate one single factor of own value above 1 (λ = 2.22) and which explains 73.9% of the variance. The three variables used have a positive force on this factor, meaning that they have the same meaning: the most anxious subjects express high scores in the three anxiety questionnaires taken into account. The pre-treatment performed was complemented by a distribution of subjects relatively to the anxiety-trait, -state, -mood variables, collected in the inclusion, through the clustering technic (K-mean) in two groups allowing to characterize an anxious group (G ANX) composed of 17 subjects and a non-anxious group (G N-ANX) composed of 47 subjects. In all cases, we considered that a difference was significant as soon as *p* < 0.05.

## Results

### Study Population

**Table 1** sums up the bio-demographic characteristics of the subjects. The night-time physiological data collected in *N* - 1 are summed up in **Table 2**. No difference was found between the trainings on each of the physiological variables. The psychological profile in the inclusion is summed up in **Table 3**. No difference is found between the trainings on the psychological variables collected.

**Table 1:** Sociodemographic characteristics of the subjects.

Mean age in year (standard deviation)	20(±2)
Military seniority (months)	1 [1 - 2]^∗^
**Educational level (%)**	
- Undergraduate studies	32
- Graduate studies	60
- Post-graduate studies	5
Reported stressfull event (%)	10
**Levels of sport (%):**	
- Recreational	49
- Regional	14
- National	10
Monthly hours of sport practice	9 [1, 75–20]^∗^


*^∗^For non-Gaussian distribution values, the description is presented by the median (first–second interquartile).*

**Table 2:** Nocturnal physiological data collected in N - 1 session.

Variables		Inclusion
Nocturnal	Mean (±SD) sleep duration (min)	407(±42)
Actimetry	Micro-awakening duration (min)	45
	Sleep efficiency index (%)	89
	Sleep fragmentation index (%)	30
	Mean (±SD) easiness to go to sleep (min)	2
Mean (±SD) nocturnal heart rate variability (LF/HF)		1.32(±0.77)


*SD, standard deviation; Min, minutes; LF/HF, low frequency/high frequency.*

**Table 3:** Psychological scores in the inclusion session (D_-1_).

Variables	*M* (±SD)
Self-esteem	32.8 (±5.5)
**Coping:**	
– Task	57.4 (±9.3)
– Avoidance	37.2 (±10.7)
– Emotion	43.7 (±12.3)
– Distraction	17.9 (±6.8)
– Social diversion	16.6 (±5.4)
**Anxiété:**	
– Trait	33.8 (±8.6)
– Etat	30.5 (±8.1)
Perceived stress	31.5 (±5.9)
**Mood:**	
– Tension/anxiety	3.1 (±3.6)
– Activity/vigor	12.3 (±5.2)
– Fatigue	2.4 (±2)
– Depression	0.7 (±1.3)
– Angor	1.3 (±2.2)
– Confusion	1.2 (±1.8)


*M (±SD), mean (±standard deviation).*

### Effect of the Commando March on the Population

All the trainees completed the commando march. The average performance was of 54.71 (±8.85) minutes. The psycho-physiological responses to the commando march show a post-challenge stress reaction. Physiologically, a post-challenge night-time sympathic activation is noticed: comparatively to *N* - 1, the LF/HF index in *N* + 1 is higher (*F* = 6.77; *p* = 0.01), and the objective sleep is of less good quality in terms of sleep quality (*F* = 11.03; *p* = 0.002), and of sleep onset latency (*F* = 6.02; *p* = 0.02). Psychologically, we notice that the anxiety-state is the highest in the D1 post-challenge (*F* = 11.54; *p* < 0.001) and that the negative moods scores are the highest [fatigue (*F* = 7.32; *p* < 0.001); depression (*F* = 7.59; *p* < 0.001); and anger (*F* = 11.48; *p* < 0.001)]. In D + 1, these scores are not different from the values collected in D - 1 and D1 pre-challenge.

### Anxious Cluster Effect in the Inclusion (G-ANX vs. G N-ANX) Onto the Psycho-Physiological Responses to the Commando March

#### Anxious Cluster Effect Onto the Inclusion Psycho-Physiological Variables

No difference is found between both groups neither in terms of average age, marital status, school level, nor in terms of hours of sports training during the previous month (*p* > 0.05). The G-ANX trainees revealed more stress events throughout their life than the G N-ANX trainees (*X*^2^= 5.45; *p* = 0.02). No difference is found in the inclusion (*N* - 1) neither on the nocturnal actimetrics variables, nor on the nocturnal heart rate variability. No difference is found between both groups in terms of performance (*p* > 0.05). **Table 4** sums up the differences observed in psychological terms between both groups.

**Table 4:** Differences observed in mean psychological scores between both groups in the inclusion session (*D*_-1_).

Variables		G ANX *M* (±SD)	G N-ANX *M* (±SD)	*p*
Population size		17	43	
Self-esteem		30.3 (±6.1)	33.8 (±5)	0.03
Coping	CISS task	54.2 (±10.9)	58.4 (±8.7)	0.15
	CISS emotion	48.2 (±9.6)	33.6 (±8.5)	0.00
	CISS avoidance	49.5 (±12)	41.5 (±11.9)	0.03
	CISS distraction	20.2 (±6.5)	17 (±6.9)	0.13
	CISS social diversion	18.2 (±6.8)	15.9 (±4.7)	0.16
Perceived stress		36.7 (±4.2)	29.4 (±5.2)	0.00
Mood	Vigor	10.9 (±6)	12.7 (±4.9)	0.24
	Fatigue	2.9 (±1.6)	2.2 (±2.1)	0.21
	Depression	1.8 (±1.8)	0.3 (±0.7)	0.00
	Anger	2.6 (±2.5)	0.8 (±1.9)	0.00
	Confusion	2.4 (±2.4)	0.6 (±1)	0.00


*G-ANX, anxious group; G N-ANX, non-anxious; *M*, mean; SD, standard deviation; CISS, Coping Inventory Stress Scale.*

#### Anxious Cluster Effects Onto the Physiological Responses of the Commando March

Regarding the LF/HF heart rate variability index, there is a session effect [LF:HF is higher at *N* + 1 (recovery) comparatively to *N* - 1; *F* = 25.68; *p* < 0.001] without any effect on the anxious status (*F* = 0.73; *p* = 0.39]. The significant interaction between the session factor and the anxiety status shows that the LF/HF index is higher at *N* + 1 for the G ANX comparatively to the G N-ANX (*F* = 13.45; *p* < 0.001). Regarding the quality of sleep, it is noticed that the fragmentation index is higher at *N* + 1 (recovery) comparatively to *N* - 1 (*F* = 8.5; *p* = 0.006). The significant interaction between the session factor and the anxiety status shows that the fragmentation index is higher at *N* + 1 for the G ANX comparatively to the G N-ANX (*F* = 5.64; *p* = 0.02).

#### Anxious Cluster Effects Onto the Psychological Responses Following the Commando March

Regarding the anxiety-state, there is an effect of the session (*F* = 5.97; *p* < 0.001) with an anxiety-state score higher at D1 post-challenge comparatively to the other sessions. There is an anxious status effect (G ANX et G N-ANX; *F* = 41.95; *p* = 0.39) with an anxiety-state score higher for the G ANX comparatively to the G N-ANX. The significant interaction between the session factor and the anxious status shows that the anxiety-state score is higher in D1 post-challenge comparatively to the other sessions for the G N-ANX (*F* = 4.17; *p* = 0.006).

Regarding the tension-anxiety mood, there is an effect of the session (*F* = 6.79; *p* = 0.002) with a tension-anxiety score higher in D+1 (recovery) comparatively to the other sessions. There is an effect of the anxious status (G ANX et G N-ANX; *F* = 18.67; *p* < 0.001) with a tension-anxiety score higher for the G ANX comparatively to the G N-ANX. No interaction between the factors is noticed (*F* = 1.75; *p* = 0.15).

Regarding the activity-vigor mood, there is an effect of the session with a lower activity-vigor score at D1 post-challenge comparatively to the other sessions (*F* = 10.09; *p* < 0.001) and an anxious status effect with an activity-vigor score lower for the G ANX comparatively to the G N-ANX (*F* = 4.02; *p* = 0.04). A trend toward an anxious session–status interaction is noticed (*F* = 2.1; *p* = 0.1). For the G ANX, the activity-vigor score tends to be stable between the sessions whereas for the G N-ANX, it is lower at D1 post-challenge comparatively to the other sessions.

Regarding the fatigue mood, there is a session effect with a higher score in D1 post-challenge comparatively to the other sessions (*F* = 10.09; *p* < 0.001), without any effect of the anxious status (*F* = 2.17; *p* = 0.14), and without any interaction between the session factor and the anxious status (*F* = 0.25; *p* = 0.85).

Regarding the depression mood, we notice that the score is higher in D1 post-challenge comparatively to the other sessions (*F* = 8.09; *p* < 0.001), and that it is higher for the G ANX comparatively to the G N-ANX (*F* = 13.06; *p* < 0.001).

Regarding the anger mood, we notice that the score is higher in D1 post-challenge comparatively to the other sessions (*F* = 9.87; *p* < 0.001) and that it is higher for the G ANX comparatively to the G N-ANX (*F* = 8.67; *p* = 0.004).

**FIGURE 2:**
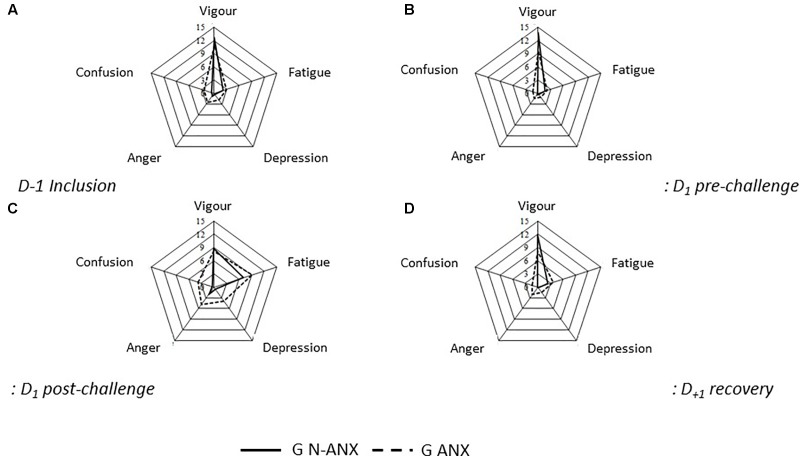
Differences between the groups over the mood variables during the different sessions. **(A)** D_-1_ inclusion, **(B)** D_1_ pre-challenge, **(C)** D_1_ post-challenge, **(D)** D_+1_ recovery. G-ANX, anxious group; GN-ANX, non-anxious group.

Regarding the confusion mood, the score is higher in D1 post-challenge comparatively to the other sessions (*F* = 11.03; *p* < 0.001). The score is higher for the G ANX (*F* = 28.75; *p* < 0.001). A trend toward an interaction is observed between the session factors and the anxious status (*F* = 2.1; *p* = 0.1). For the G N-ANX, the confusion score tends to be stable between the sessions whereas for the G ANX, it is higher in D1 post-challenge comparatively to the other sessions. **Figure 2** sums up the differences between the groups over the mood variables during the different sessions (D-1, D1 pre-challenge, D1 post-challenge, and D+1).

## Discussion

The purpose of this study was to assess the reaction to stress induced by a sports challenge with a strong commitment and the recovery the day after the challenge according to the subjects’ anxious status. First, it confirmed the stressor role of the selecting challenge played by the commando march. The increase of the activation psychological markers (anxiety, anger moods) was associated with physical fatigue (fatigue mood) and mental fatigue (depression mood). The psychological recovery was observed the very next day after the exercise. The night that followed the exercise allowed to assess the recovery. A night-time parasympathetic rebound (Myllymaki et al., 2012) and a quality of sleep with few awakenings reflect the quality of the anabolic response of the sleep in the post-exercise recovery (Schall et al., 2013). The results show that the sleep was not restorative as shown by the night-time heart rate variability and the deterioration of the sleep quality. These observations contrast with the data showing that the isolated or regular physical exercise practice is an important factor to promote the sleep (Youngstedt et al., 1999). The disruption of the post-exercise night-time recovery is all the more important to consider that the subjects enrolled in the study are characterized by a protective psychological profile: high level of self-esteem and low level of anxiety-trait. The adjustments strategies show an adaptive pattern relatively to the general population, the subjects being characterized by a high level of on-task coping, and a moderate level of emotional coping.

Second, in our study, the population was segregated into two subgroups according to the anxious status and by taking into account both the anxious personality and the dynamic process induced by the commando march (anxiety-state and -mood). This segregation showed that the subjects of the anxious group were characterized by a lower level of self-esteem and a higher level of coping focused on emotion and avoidance. It also had implications on the stress response at different steps of the exercise. Before the challenge, the anxious subjects expressed a higher level of perceived stress as well as an increase of the activation psychological markers (anxiety, anger moods) and of mental fatigue (depression and confusion moods) for a same level of physical fatigue (fatigue mood). If this state reflects an anticipatory anxiety in relation to previous experiences, this reactivity before the exercise raises questions the memorization of previous physical exercises (Stranahan et al., 2008) and the way he anticipates the level of constraint (Dishman et al., 2000). A neurobiological profile with an increase of reactivity to a stressor was put forward (Pruessner et al., 2005). It combines a reduction of the hippocampus volume in a functional MRI, an increase of the reactivity of the corticotropic axis, and a reduction of the self-esteem. The pertinence of this profile requests to be assessed within the frame of an excessive reaction to stress when facing a sport event with a strong commitment. Immediately after the exercise, the anxious status was characterized by the maintenance of the psychological markers of activation (anxiety, anger) and of mental fatigue (depressive and confused mood) at a higher level than the non-anxious status. However, the anxious status did not have any impact neither onto the physical performance nor onto the fatigue.

Lastly, the night-time recovery is of less good quality in the anxious subjects than in the subjects with a non-anxious status. Hughes et al. (2006) have shown that the existence of depressive symptoms were slowing down the recovery after an exercise. Thus, the stress activation excess in peri-exercise was associated with an anomaly of its extinction in the recovery phase. In the end, these data suggest that in subjects with an anxious status, practicing an implying activity generates an inadequate stress reaction. This excessive reactivity concerns on the one hand the modalities of sports practice in the treatment of anxiety. The physical activity modalities in these treatments must focus on promoting a practice without generating neither an internal nor an external obligation in the anxious subjects. On the other hand, the excessive reactivity questions in the longer term about the consequences of a repeated and constraint physical activity onto these subjects’ psychological and physical health. In particular, it questions the role that the anxious status could have in the risk of emergence of an overtraining syndrome. This syndrome, which is characterized by a performance level decrement without any modification of the investment in the sports practice, is clinically expressed in very different ways: anxiety, depression, fatigue, anger, lack of confidence, insomnia (Kentta and Hassmen, 1998). The absence of any clinical sign pathognomonic for an overtraining state, is associated with a difficulty to extract specific biological signs from it (Lee et al., 2017). However, the repetition of excessive stress reactions was put forward as contributing to a risky sports practice (Angeli et al., 2004). The repeated stress would induce a functional problem likely to lead to a dysfunction. The overtraining could be considered as a stress pathology expressing the impossibility for an individual to manage his stress response to the minimum required by the demand. These costs fall within the framework of the allostasis theory, which characterizes the recovery process or not of the homeostasis in the presence of constraint (Charney, 2004). The evaluation of the anxious status would allow to improve the detection of subjects at highest risk complementary to the overtraining questionnaire. The latter does not allow the systematic detection of the most motivated subjects who are the most vulnerable (Maso et al., 2005): these subjects, who barely listen to the body alarm signals, will meet any decrease of performance by an increase of training. This description fits in with the absence of impact of the anxious status onto the physical fatigue experienced after the commando march. Taking this status into account could be necessary to determinate the training programs for regular sportsmen. These data are all the more important that the consequences of overtraining can be dramatic for a sports career.

This study has shown two main limitations. Even though no difference in the main variables was observed over the four training sessions organized in different seasons, an impact of the seasonality onto the mood cannot be excluded (Kurlansik and Ibay, 2012). Then, the study has only included male subjects, which implies reproducing these results in a female population.

## Conclusion

The commando march constitutes a military paradigm of sport under constraint that can be transposed to the civilian environment within the frame of a selective or competitive sports practice. The results obtained have applications for the health of both civilian and military sportsmen. They clearly raise the question of taking into account the anxiety in the sports practice programming. When the anxiety is taken into account, the modalities must detect any practice under constraint. For regular sportspeople, the modalities must integrate the anxious status in the programs of the sessions’ repetition. These results highlight the interest for coupling the physical activity to stress and anxiety management techniques whether on the occasional sportsman, the regular sportsman or the competitor.

## Author Contributions

ES, ZF, FC, and MT were involved in the conception and trial design. GT, ES, CM-K, and MT wrote the draft of the article. ES, ZF, FC, CM-K, and MT contributed to the refinement of the study protocol and provided expert insight. ES and FC were responsible for the ethics committee. All the authors were involved in final approval of the manuscript.

## Conflict of Interest Statement

The authors declare that the research was conducted in the absence of any commercial or financial relationships that could be construed as a potential conflict of interest. The reviewer RV declared a past co-authorship with one of the authors CM-K to the handling Editor.
